# Aggresomes do not represent a general cellular response to protein misfolding in mammalian cells

**DOI:** 10.1186/1471-2121-9-59

**Published:** 2008-10-20

**Authors:** Simon Beaudoin, Kevin Goggin, Cyntia Bissonnette, Catherine Grenier, Xavier Roucou

**Affiliations:** 1 Department of Biochemistry, Faculty of Medicine, University of Sherbrooke, Sherbrooke, Québec J1H 5N4, Canada

## Abstract

**Background:**

Aggresomes are juxtanuclear inclusion bodies that have been proposed to represent a general cellular response to misfolded proteins in mammalian cells. Yet, why aggresomes are not a pathological characteristic of protein misfolding diseases is unclear. Here, we investigate if a misfolded protein inevitably forms aggresomes in mammalian cells.

**Results:**

We show that a cytoplasmic form of the prion protein may form aggresomes or dispersed aggregates in different cell lines. In contrast to aggresomes, the formation of dispersed aggregates is insensitive to histone deacetylase 6 inhibitors and does not result in cytoskeleton rearrangements. Modulation of expression levels or proteasome inhibitors does not alter the formation of dispersed aggregates.

**Conclusion:**

Our results establish that aggresomes are not obligatory products of protein misfolding in vivo.

## Background

The deposition of protein aggregates is a pathological feature of a large number of diseases targeting the nervous system and/or peripheral organs. Neurodegenerative diseases include Alzheimer's disease (AD), Parkinson's disease (PD), Huntington's disease (HD) and related polyglutamine disorders, amyotrophic lateral sclerosis (ALS), and prion diseases, [1-3]. Besides the brain, other organs affected in aggregation disorders include the liver and/or the lung in alpha1-antitrypsin deficiency and cystic fibrosis, and the heart in familial amyloid cardiomyopathy [4].

In order to elucidate the relationship between protein aggregation and cell dysfunction, protein aggregation has been recapitulated in cultured cells by overexpressing wild-type or mutant proteins. These proteins are alpha-synuclein and parkin in PD [5,6], huntingtin in HD [7], presenilin1 and presenilin-binding proteins in AD [8], polyglutamine-containing proteins in polyglutamine diseases [9], superoxide dismutase in ALS [10], the prion protein (PrP) in prion diseases [11,12], cystic fibrosis transmembrane conductance regulator in cystic fibrosis [13]. These studies have defined several features including the coalescence of protein deposit at the centrosome, and the collapse of intermediate filament vimentin protein forming a cage around the deposits. Such juxtanuclear protein deposits were termed aggresomes, and it was originally proposed that aggresome formation is a general cellular response to the accumulation of misfolded proteins [13].

There is recent evidence that protein aggregates in animal models of human neurodegenerative diseases resemble aggresomes. Mutant superoxide dismutase molecules form aggresome-like particles in a mouse model of ALS [10]. Prion-infected mice also produce perinuclear aggresomal-like particles [14]. Moreover, it was proposed that Lewy-bodies formation in PD patients is similar to the formation of aggresomes in cultured cells [15,16]. However, aggresomes are not a key pathological feature of all neurodegenerative diseases in humans, which suggests that they may not represent a general response to protein misfolding in vivo.

We have previously reported that a cytoplasmic form of PrP termed CyPrP forms aggresomes in murine N2a and human embryonic kidney 293 cells [12]. In the present study, we have characterized the cellular and molecular response to CyPrP expression in various cells. Our data indicate that although CyPrP misfolds and produces insoluble particles in all cell lines tested, aggregates display two types of molecular morphology. We confirmed that CyPrP spontaneously forms aggresomes in N2a cells. By contrast, other cells including Hela, Cos-7, Huh-7 cells exclusively produce dispersed aggregates which are not juxtanuclear and are not associated with a cage-like structure of vimentin. These findings lead us to propose that cellular management of protein misfolding is complex, and that aggresomes are not obligatory end-products of protein misfolding in cells.

## Methods

### Cell culture, transfections and treatment

Human cervical cancer HeLa, embryonic kidney 293, mammary adenocarcinoma MCF-7, mouse neuroblastoma N2a, fibroblasts NIH3T3, and monkey fibroblasts COS-7 were cultured in Dulbecco's modified Eagle medium (DMEM) supplemented with 10% FBS. Transfections were conducted with exponentially growing cells using Lipofectamine 2000 as described by the manufacturer (Invitrogen).

For tetracycline-regulated expression, cells were transfected with pRevTet-On (Invitrogen) and selected with 200 μg/ml G418 (Sigma) to obtain individual clones. Selected clones were propagated and transfected with pRevTRE-CyPrP^EGFP^. Cells were then tested for their tetracycline-regulated CyPrP^EGFP ^expression by western blotting. For inhibition of histone deacetylase, cells were transfected with CyPrP^EGFP ^and incubated for 24 h in the presence of 5 μM scriptaid or its inactive structural analog nullscript (BioMol), or in the presence of 5 μM tubacin or its inactive structural analog (kindly provided by Dr Ralph Mazitschek, Broad Institute, Cambridge, MA, USA).

### Plasmid constructs and antibodies

Cloning of CyPrP^EGFP^, a form of PrP without N-terminal secretion and C-terminal glycosylphosphatidylinositol anchor signal peptides was described previously [12]. CyPrP^DsRed2 ^was obtained from PrP^DsRed2 ^[12], by deleting the N- and C-terminal signal peptides. DsRed2, like EGFP, was introduced in the natural SmaI restriction site of human gene encoding PrP, *Prnp*, at bp113 (amino-acid 38). To construct CyPrP^mOrange^, mOrange was amplified as a Sma1 fragment from pREST-BmOrange kindly provided by Dr Roger Tsien (University of California San Diego, CA, USA). The PCR product was introduced in the natural SmaI restriction site of *Prnp*. A clone containing mOrange in the right orientation was selected. CyPrP124stop^mOrange ^was obtained by inserting the PCR product in the SmaI restriction site of CyPrP124stop [12]. For tetracycline-regulated expression of CyPrP^EGFP^, CyPrP^EGFP ^was cloned between the BamH1 and HindIII restriction sites of pRevTRE (Invitrogen). All clones were sequenced and their expression verified by western blot analysis.

The construct encoding GFP-250 was kindly provided by Dr Elisabeth Sztul (University of Alabama at Birmingham, AL USA).

Monoclonal anti-PrP (clone 3F4), anti-tubulin-alpha (clone 236–10501), and anti-vimentin (clone V9) antibodies were purchased from Chemicon, Molecular Probes, and Sigma, respectively.

### Biochemical assays

To fractionate protein aggregates on sucrose gradient, cells from a 10 cm-petri dish were harvested, washed twice with ice-cold PBS, and resuspended in 2 ml hypotonic fractionation buffer (KCl 10 mM; β-mercaptoethanol 1 mM; MgCl2 1.5 mM; Hepes 10 mM, pH7.9). Cells were mechanically broken with 20 strokes in a Dounce homogenizer. Nuclei and unbroken cells were removed by centrifugation 5 min at 1,500 × g. The supernatant was centrifuged 5 min at 10,000 × g to pellet the aggregates. The pellet was resuspended with 0.5 ml fractionation buffer and layered on top of a continuous 1.2 M-2 M sucrose gradient in fractionation buffer. After a 2 h centrifugation at 150,000 g in a Beckman SW60 Ti rotor at 4°C, 1 ml fractions were collected and proteins were precipitated with 12% trichloroacetic acid. The resulting pellets were dissolved in sample buffer, separated by SDS-PAGE and analyzed by immunoblotting.

To assay the detergent insolubility of PrP, cells were solubilized in lysis buffer (0.5% sodium deoxycholate, 0.5% Triton X-100, 150 mM NaCl, and 50 mM Tris-HCl, pH 7.5) supplemented with protease inhibitors(mini-tablets from Roche). The lysate was clarified by centrifugation at 16,000 × *g *for 3 min, followed by ultracentrifugation at 186,000 × *g *for 40 min. The pellet from the second centrifugation, containing detergent-insoluble PrP, was resuspended in SDS-PAGE sample buffer. Detergent-soluble PrP was precipitated from the supernatant by addition of 0.05 volumes of 10% SDS and 4 volumes of methanol at -20°C. PrP was visualized by SDS-PAGE and Western blotting using antibody 3F4 and ECL detection [12].

### Immunofluorescence and fluorescence in situ hybridization

Cells grown on coverslips were fixed and processed for immunofluorescence using anti-PrP monoclonal antibodies 3F4 as previously described [12]. For in situ staining, permeabilized cells were incubated 10 min with 2 × SSC, and hybridized with 1 nM of an end-labeled biotinylated oligo-dT (50 nucleotide, IDT) overnight at 40°C. After washing twice with 2 × SSC and once with 0.5 × SSC, cells were equilibrated in 1 × PBS containing 1 mg/ml BSA. Cells were incubated with 2 μg/ml Alexa Fluor 633-labeled strepavidin (Molecular Probes) in 1 × PBS containing 1 mg/ml BSA. After a 1 h incubation, cells were washed and mounted as previously described [12]. For fluorescence analysis, cells were examined with an Eclipse TE2000-E visible/epifluorescence inverted microscope (Nikon Corporation) equipped with band pass filters for fluorescence of Hoechst (Ex. D340/40: Em. D420), GFP (Ex. D450/40: Em. D500/50) and tetramethylrhodamine isothiocyanate (TRITC) (Ex. D528/25: Em. D590/60) (Nikon Corporation). Photomicrographs of 1344 × 1024 pixels were captured using a 100 × oil immersion objective and Orca cooled color digital camera (Hamamatsu Photonics). Images were processed using NIS Elements AR software (Nikon Corporation). Within the same figure, all pictures were taken with identical exposure time except in figure 3.

### Metabolic labelling

10^6 ^cells were transfected either with the empty vector, EGFP or CyPrP^EGFP ^and incubated 24 hours at 37°C. Cells were washed twice with PBS and incubated 20 minutes in starvation media (DMEM without methionine and cysteine, Gibco). Cells were then pulsed with 25 μCi/ml ^35^S labelling mix (Easy Tag Express protein labelling mix, NEG772, Perkin-Elmer) for 1 h at 37°C. In control experiments, the translation inhibitor cycloheximide was added at 30 μg/ml prior to starvation. After labelling, cells were washed twice with PBS, scraped, collected in 1 ml PBS and centrifuged 5 minutes at 5000 rpm at 4°C. Pellets were lysed with 100 μl of buffer B [10 mM Tris pH 8.0, 100 mM NaCl, 0,5% Nonidet-P40, 0,5% Sodium deoxycholate and 1 mini EDTA-free protease inhibitor tablet (Roche) per 10 ml]. Proteins were dosed with BCA protein assay (Pierce) and 50 μg of total proteins were loaded on 10% polyacrylamide denaturing gels. Gels were then exposed 24 hrs to a Phosphor screen (GE Healthcare) and scanned on a Storm 860 Imager (Molecular Dynamics).

## Results and discussion

**Figure 1:**
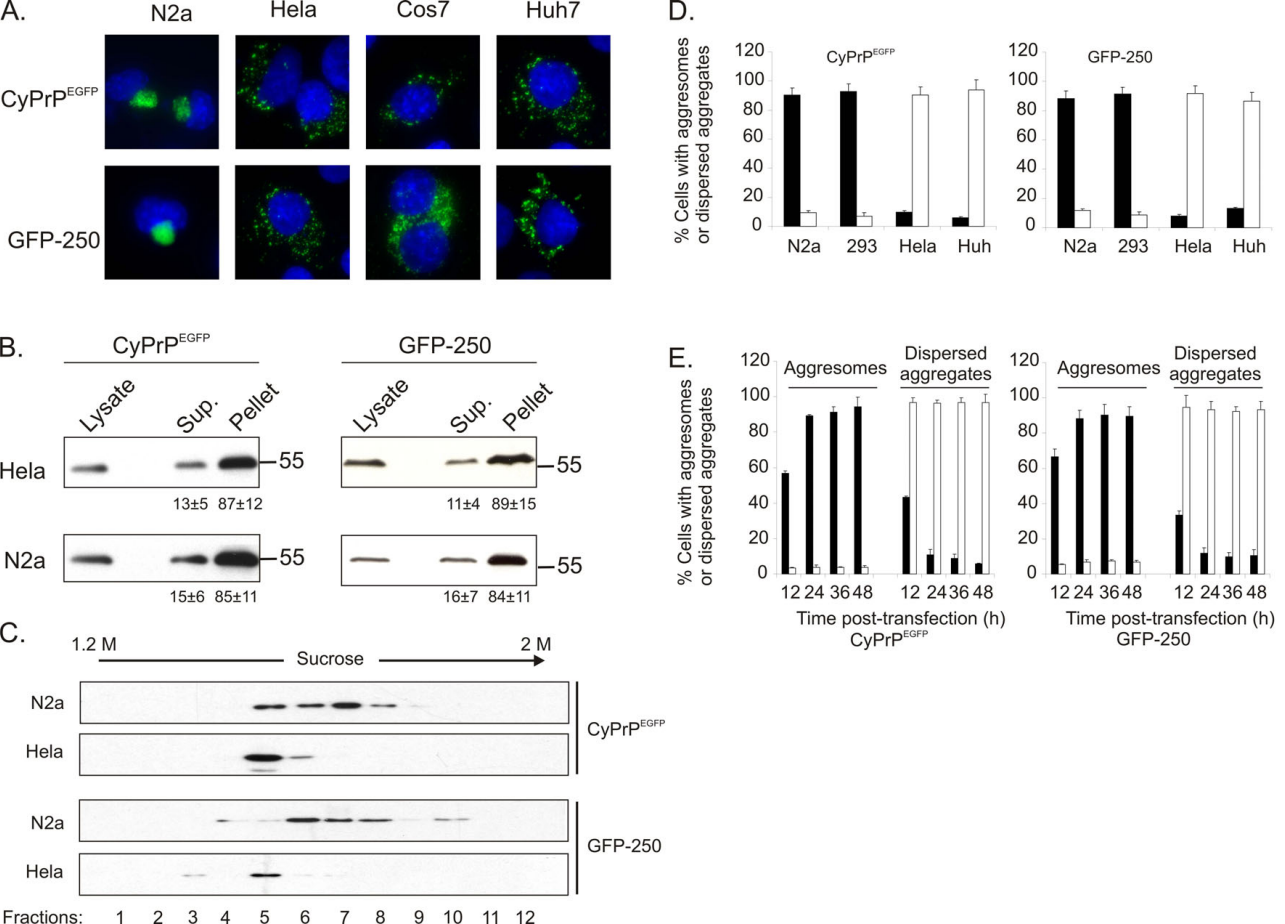
**Characterization of two types of CyPrP^EGFP ^aggregates in different cells**. (A) The cellular localization of CyPrP^EGFP ^and GFP-250 was visualized after 24 h of expression in N2a, Hela, Cos7, and Huh-7 cells. Nuclei were stained with Hoechst (blue). Green and blue channels are shown merged. (B) Western blot of CyPrP^EGFP ^and GFP-250 in 100 μg of protein extract from detergent-soluble (*Sup*.) and -insoluble (*Pellet*) fractions of N2a and Hela cells. The percentage of protein in the supernatant and pellet indicated below each blot was measured by densitometric analysis, and represents the mean and S.D. of three independent experiments. (C) Western blot of CyPrP^EGFP ^and GFP-250 in different fractions of a 1.2 to 2 M sucrose gradient loaded with lysates from N2a or Hela cells. This experiment is representative of two independent experiments. (D) The percentage of cells displaying aggresomes (black columns) or dispersed aggregates (white columns) was calculated in N2a, HEK293, Hela, and Huh-7 cells 24 h post-transfection. (E) The percentage of N2a (black columns) and Hela (white columns) cells displaying aggresomes or dispersed aggregates was calculated different times post-transfection with CyPrP^EGFP ^or GFP-250. (D, E) Data represent the mean and S.D. of three independent experiments. More than 200 cells were counted for each condition.

To test if aggresomes formation is a general cellular response to protein misfolding, different cells were transfected with a construct encoding a cytoplasmic form of prion protein genetically fused to EGFP, termed CyPrP^EGFP^. As previously described, CyPrP^EGFP ^formed aggresomes in mouse neuroblastoma N2a cells (Fig. 1A) [12]. In contrast, CyPrP^EGFP ^produced dispersed aggregates in other cells, including Hela, COS-7, Huh-7, MCF-7, and NIH3T3 (Fig. 1A; not shown). We compared the detergent solubility of CyPrP^EGFP ^in N2a and Hela cells (Fig. 1B). After ultracentrifugation of detergent lysates, CyPrP^EGFP ^was largely insoluble, with more than 80% of the protein found in the pellet of N2a and Hela lysates. The difference of density of both types of aggregates was analysed in a sucrose gradient. Aggresomes were detected in fractions 5 to 8, whilst dispersed aggregates were present in fraction 5 (Fig. 1C).

It was of interest to test the generality of these findings with another aggresome-forming protein. To address this point, we expressed a cytosolic chimera termed GFP-250. This chimeric polypeptide composed of the entire soluble protein GFP fused at its COOH terminus to a 250-amino acid fragment of the cytosolic protein, p115, has been used to determine the dynamics of aggresomes formation [17]. Similar to CyPrP^EGFP^, GFP-250 formed aggresomes in N2a cells and dispersed aggregates in Hela, COS-7, and Huh-7 cells (Fig. 1A). The solubility of GFP-250 was independent of the formation of aggresomes or dispersed aggregates, and the protein was largely insoluble in N2a or Hela cells (Fig. 1B). Like CyPrP^EGFP^, GFP-250 aggresomes and dispersed aggregates also displayed differences of density in a sucrose gradient (Fig. 1C).

We determined the proportion of aggresomes and dispersed aggregates 24 h after transfection (Fig. 1D). N2a and 293 cells produced a majority of aggresomes, while the proportion of dispersed aggregates was largely dominant in Hela and Huh-7 cells. One possibility to explain that Hela and Huh-7 cells did not produce aggresomes after 24 h of expression could be that the kinetics of coalescence of individual aggregates at the centrosome are slower in these cells. To address this question, we determined the proportion of cells producing aggresomes and dispersed aggregates different times post-transfection. After 12 h of transfection, cells produced mostly dispersed aggregates (Fig. 1E). At 24 h, N2a cells mainly produced aggresomes, indicating that dispersed aggregates had coalesced at the centrosome (Fig. 1E). In contrast, over 80% of Hela cells displayed dispersed aggregates even 96 h post-transfection (Fig. 1E). Inhibiting proteasomes accelerate the formation of perinuclear aggresomes [14]. Yet, two proteasome inhibitors, epoxomycin and MG132, did not induce the coalescence of dispersed aggregates in a perinuclear aggresome (not shown). From these data, we conclude that end-products of misfolded proteins are aggresomes in N2a cells, and dispersed aggregates in Hela cells. Since similar results were obtained using CyPrP^EGFP ^and GFP-250, the following experiments were carried out with CyPrP^EGFP^.

**Figure 2:**
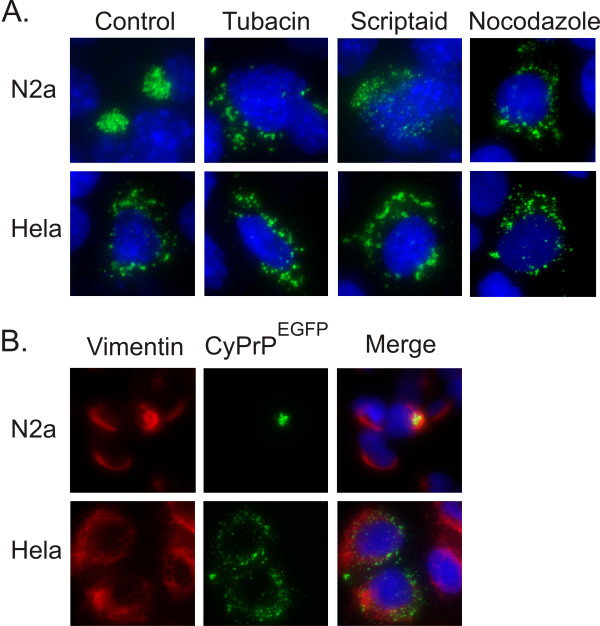
**The formation of dispersed aggregates is independent of deacetylase HDAC6, microtubules, and intermediate filament vimentin**. (A) The cellular localization of aggregates was determined in N2a and Hela cells transfected with CyPrP^EGFP ^for 24 h in the absence (control) or in the presence of 5 μM tubacin, 5 μM scriptaid, or 5 μM nocodazole. Nuclei were stained with Hoechst (blue). (B) Immunofluorescence analysis of vimentin (red) in N2a and Hela cells transiently transfected with CyPrP^EGFP^. Nuclei were stained with Hoechst (blue). Red (left panel) and green (middle panel) channels are shown separately, and merged with blue channel (right panel).

The next experiments were designed to determine whether dispersed aggregates may represent independent aggresomal particles. The microtubule-associated histone deacetylase6 (HDAC6) regulates the formation of aggresomes by recruiting misfolded proteins to dynein motors on microtubules, and inhibition or down-regulation of HDAC6 prevents the assembly of aggresomes [18]. Therefore, it was important to test the effect of HDAC6 inhibitors on the assembly of dispersed aggregates. Tubacin and scriptaid, two specific HDAC6 inhibitors [19,20], efficiently prevented the formation of aggresomes in N2a cells (Fig. 2A). Inactive structural analogs of tubacin and scriptaid, niltubacin and nullscript, respectively had no effect (not shown). In Hela cells, both tubacin and scriptaid did not alter the formation of dispersed aggregates (Fig. 2A). We concluded that HDAC6 is not involved in the formation of dispersed aggregates. Aggresome formation requires an intact microtubule network [13]. The absence of CyPrP^EGFP ^aggresomes in Hela cells may result from a different organization of the microtubule network in these cells. Alternatively, the formation of dispersed aggregates may be independent from microtubules. This second hypothesis is supported by our previous observation that the microtubule-associated HDAC6 is not involved in the assembly of dispersed aggregates (Fig 2A). Thus, in a second set of experiments, we evaluated the effect of the microtubule-depolymerising agent nocodazole. Nocodazole did not prevent the formation of dispersed aggregates (Fig. 2A). In a control experiment, we ascertained that nocodazole prevented the assembly of aggresomes in N2a cells as previously described (Fig 2A) [12]. This result demonstrates that the formation of dispersed aggregates does not require an intact microtubule network. In a third set of experiments, we determined the distribution of intermediate filament vimentin protein which forms a cage surrounding aggresomes [13]. The concentration of vimentin filaments around CyPrP^EGFP ^aggresomes was confirmed in N2a cells (Fig. 2B). In sharp contrast, the distribution of vimentin remained unchanged in Hela cells (Fig. 2B). All together, these results indicate that dispersed aggregates are not small independent aggresomal particles.

**Figure 3:**
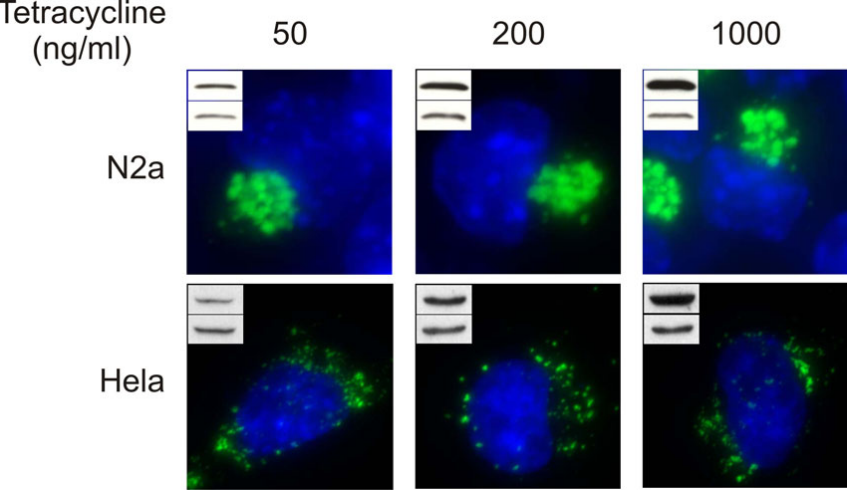
**The assembly of aggresomes or dispersed aggregates is independent of the level of expression of CyPrP^EGFP^**. Aggregates were visualized by fluorescence 24 h after addition of different concentrations of tetracycline in the culture medium of N2a and Hela cells. From left to right, exposure times for the green channel are 850 ms, 400 ms, and 60 ms. Insets represents western blot analysis of CyPrP^EGFP ^(top band) or tubulin to check for equal loading (bottom band). Results are representative of 3 independent experiments.

We tested if differences in expression levels could explain the formation of aggresomes or dispersed aggregates. N2a and Hela cells were transfected with tetracycline-regulated expression vectors. In this experimental paradigm, level of CyPrP^EGFP ^is modulated by adding to the cells increasing concentrations of tetracycline. Even at very low levels of expression CyPrP^EGFP ^formed aggresomes in N2a cells (Fig. 3). The formation of dispersed aggregates in Hela cells was also independent of CyPrP^EGFP ^expression levels (Fig. 3).

**Figure 4:**
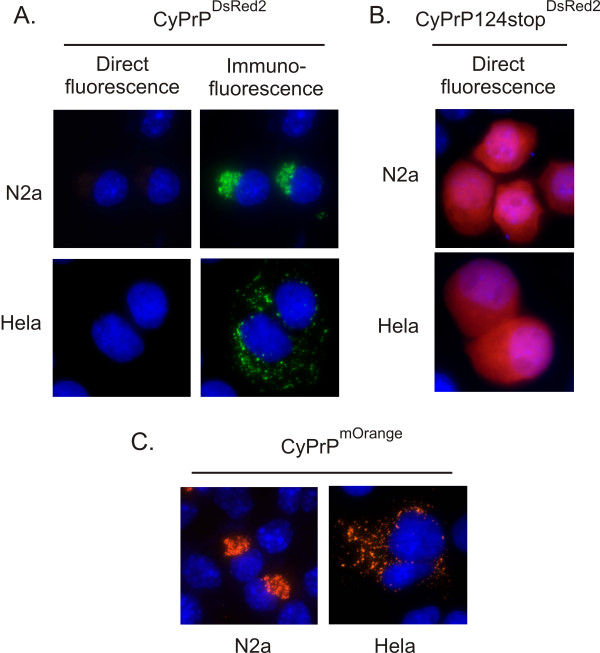
**Oligomeric DsRed2 does not fluoresce when genetically fused to CyPrP**. (A) Direct fluorescence or immunofluorescence (3F4 anti-PrP antibodies, green) of N2a and Hela cells expressing CyPrP^DsRed2^. Nuclei were stained with Hoechst (blue). Blue and red channels are shown merged. (B, C) Direct fluorescence of N2a and Hela cells expressing CyPrP124stop^DsRed2 ^(B) or CyPrP^mOrange ^(C). Nuclei were stained with Hoechst (blue). Blue and red channels are shown merged.

Since the size and location of CyPrP^EGFP ^aggregates in N2a and Hela cells is different, one could suggest that the molecular assembly of proteins inside these aggregates is also different. To test this hypothesis, we took an approach based on monomeric and oligomeric fluorescent proteins. The EGFP moiety fused to CyPrP is fluorescent, indicating that structural constraints on individual CyPrP^EGFP ^molecules within aggregates are limited and that EGFP is correctly folded. However, more constraints may be imposed on the arrangement of molecules inside the aggregates. Therefore, we reasoned that CyPrP fused to an obligate oligomeric fluorescent protein may not necessarily produce fluorescent aggresomes and/or dispersed aggregates. We used DsRed2, an obligate tetramer fluorescent protein [21]. Surprisingly, CyPrP^DsRed2 ^did not form fluorescent aggregates neither in N2a cells, nor in Hela cells (Fig. 4A). This result indicated that DsRed2 cannot form tetramers when fused to CyPrP. We performed several control experiments to validate this result. First, CyPrP^DsRed2 ^aggregates were detected by immunofluorescence, indicating that DsRed2 does not inhibit the aggregation of CyPrP (Fig. 4A). Second, we expressed CyPrP^DsRed2^124stop, a cytoplasmic form of PrP unable to form aggregates [12]. CyPrP^DsRed2^124stop produced diffuse red fluorescence in the cytoplasm (Fig. 4B), indicating that DsRed2 fused to a cytoplasmic soluble form of PrP is able to form fluorescent tetramers. Third, we engineered a fusion protein between CyPrP and mOrange, a fluorescent monomeric version of DsRed2 [22]. N2a and Hela cells expressing CyPrP^mOrange ^displayed fluorescent aggregates (Fig. 4C). All together, these data indicate that the arrangement of CyPrP molecules in aggresomes or dispersed aggregates is such that CyPrP^DsRed2 ^cannot assemble into fluorescent tetramers. Furthermore, the molecular assembly of CyPrP is likely similar in both aggresomes and dispersed aggregates.

**Figure 5:**
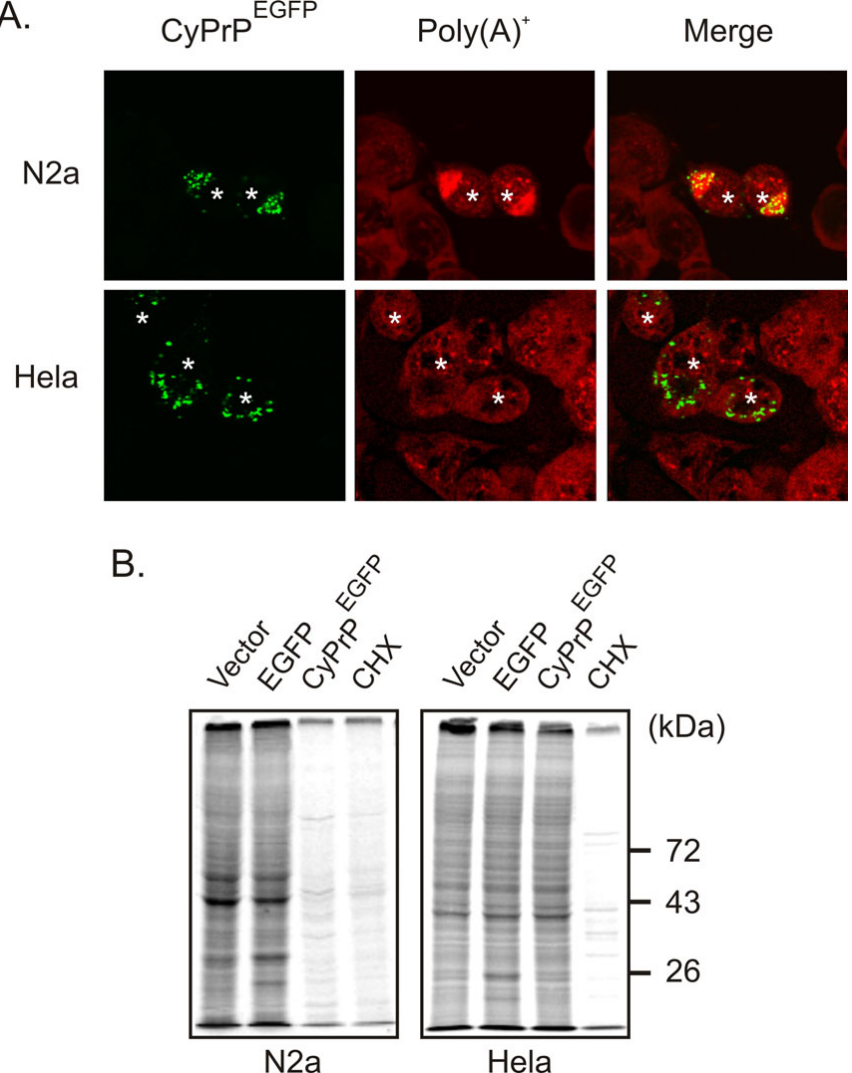
**In contrast to aggresomes, dispersed aggregates of CyPrP^EGFP ^do not affect the localization of poly(A)^+^-RNA or protein translation**. (A) N2a and Hela cells were transiently transfected with CyPrP^EGFP^. After 24 h, cells were fixed, permeabilized, and processed for in situ hybridization to detect poly(A)^+ ^RNA (Red), and analysed by fluorescence microscopy. Asterisks indicate transfected cells. (B) Total protein production was measured by analyzing newly synthesized proteins in N2a and Hela cells transfected with empty vector, EGFP, or CyPrP^EGFP^, as indicated. Cells were labelled with 25 μCi/ml ^35^S labelling mix in methionine (Met)- and cysteine (Cys)-deficient medium. Where indicated, medium contained 30 μg/ml cycloheximide (CHX). Equal amounts of proteins were separated by 10% SDS-PAGE and radioactivity signals were determined with a phosphorimager. The position of the molecular mass markers is indicated on the right.

Besides structural differences between aggresomes and dispersed aggregates, it was of interest to test for functional dissimilarities. CyPrP aggresomes induce the aggregation of poly(A^+^) RNA, resulting in the shutoff of protein translation [23]. We investigated whether dispersed CyPrP aggregates in Hela cells also induce the aggregation of poly(A)^+ ^RNA and a decrease in protein synthesis. In untransfected cells, poly(A)^+ ^RNA displayed a punctuate and diffuse staining and was distributed between the nucleus and the cytoplasm of N2a and Hela cells (Fig. 5A). In cells expressing CyPrP^EGFP^, we observed the aggregation of poly(A)^+^-RNA in N2a cells, whilst the distribution of poly(A)^+^-RNA remained unchanged in Hela cells (Fig. 5A). Figure 5B shows that CyPrP^EGFP ^expression largely reduced levels of protein translation compared to mock-transfected cells or cells expressing the control cytoplasmic EGFP protein in N2a cells (Fig. 5B). In contrast, there was no decrease in protein translation in Hela cells expressing CyPrP^EGFP ^(Fig. 5B). In control experiments, protein synthesis was inhibited with cycloheximide. These results indicate that aggresomes and dispersed aggregates have different impacts on the physiology of the cells.

Protein aggregation is a major characteristic of most neurodegenerative diseases [2,3]. In order to elucidate the relationship between protein aggregation and cell dysfunction, in vitro models using recombinant proteins have been developed. The reconstitution of protein aggregation in cultured cells has led to the proposition that aggresomes are a general response to protein misfolding [13]. In this report, we show that CyPrP may either form aggresomes or dispersed aggregates independently of protein levels and of proteasomal activity. This result is significant since it demonstrates that aggresome is not an obligatory final product of protein misfolding, and it may explain in part why aggresome is not a conspicuous pathological feature of all neurodegenerative diseases in humans.

Several lines of evidence indicate that the mechanisms involved in the assembly of cytoplasmic protein aggregates are more complex than a simple accumulation of misfolded proteins. First, the observation that CyPrP does not form aggresomes in some cell lines, including Cos-7, Hela, and Huh-7 may indicate that such cells are not able to assemble aggresomes. Yet, Cos-7 has been used as a cell model to investigate the dynamics of aggresome formation [24]. Similarly, several proteins are able to form aggresomes in Hela and Huh-7 cells [25-27]. Therefore, all cells probably possess the molecular machinery to form aggresomes. However, CyPrP^EGFP ^does not accumulate in an aggresome in all cells. Second, in contrast to monomeric EGFP and mOrange fluorescent proteins, the obligate tetrameric DsRed2 protein does not produce fluorescence when fused to CyPrP. This observation suggests that structural constraints imposed on individual molecules within aggregates are important and that the arrangement of protein molecules may not be random. Third, in contrast to other proteins or other mutant forms of PrP, the formation of CyPrP aggresomes is independent of expression levels and proteasome inhibition [11,13]. Furthermore, proteasome inhibition does not result in the conversion of dispersed aggregates into aggresomes. Forth, by co-expressing two unrelated misfolded proteins, it was observed that nonspecific co-aggregation between hydrophobic proteins does not occur and that protein aggregation is highly specific [28].

## Conclusion

Our data using HDAC6 inhibitors and nocodazole indicate that dispersed aggregates do not require the microtubules network to assemble. A possible interpretation of these observations is that dispersed aggregates represent intermediate particles in an incomplete aggresomal pathway in Cos-7, Hela, and Huh-7 cells. Why dispersed aggregates are not transported to and accumulated at the centrosome in these cells remains to be determined. Another interpretation is that dispersed aggregates form through a novel pathway independent from the aggresomal pathway.

Consequently, there is accumulating evidence that protein aggregation in vivo cannot be considered to result from the simple coalescence of misfolded proteins, driven by interactions between solvent-exposed hydrophobic surfaces that are normally buried. The elucidation of the mechanisms governing protein aggregation, assembly and cellular localization of aggregates will help understanding aggregation diseases including a majority of neurodegenerative disorders.

## Abbreviations

CyPrP^EGFP^: cytoplasmic prion protein genetically fused to EGFP; HDAC6: histone deacetylase 6.

## Authors' contributions

SB performed performed the experiments in Fig 1DE, 2, 3, 4, and 5A. KG performed the experiments in Fig 1 and Fig 5B. CB established the stable cell lines used in Fig 3. CG provided general assistance for cloning all DNA constructs and for in situ hybridization experiments. XR conceived of the study, participated in its design and coordination and helped to draft and edit the manuscript. All authors read and approved the final manuscript.

## References

[B1] Kakizuka A (1998). Protein precipitation: a common etiology in neurodegenerative disorders. Trends Genet.

[B2] Soto C (2003). Unfolding the role of protein misfolding in neurodegenerative diseases. Nat Rev Neurosci.

[B3] Ross CA, Poirier MA (2004). Protein aggregation and neurodegenerative diseases. Nat Med.

[B4] Ellisdon MA, Bottomley SP (2004). The role of protein misfolding in the pathogenesis of human diseases. IUBMB Life.

[B5] Masliah E, Rockenstein E, Veinbergs I, Mallory M, Hashimoto M, Takeda A, Sagara Y, Sisk A, Mucke L (2000). Dopaminergic loss and inclusion body formation in alpha-synuclein mice: implications for neurodegenerative disorders. Science.

[B6] Junn E, Lee SS, Suhr UT, Mouradian MM (2002). Parkin accumulation in aggresomes due to proteasome impairment. J Biol Chem.

[B7] Waelter S, Boeddrich A, Lurz R, Scherzinger E, Lueder G, Lehrach H, Wanker EE (2001). Accumulation of mutant huntingtin fragments in aggresome-like inclusion bodies as a result of insufficient protein degradation. Mol Biol Cell.

[B8] Namekata K, Nishimura N, Kimura H (2001). Presenilin-binding protein forms aggresomes in monkey kidney COS-7 cells. J Neurochem.

[B9] Taylor JP, Tanaka F, Robitschek J, Sandoval CM, Taye A, Markovic-Plese S, Fischbeck KH (2003). Aggresomes protect cells by enhancing the degradation of toxic polyglutamine-containing protein. Hum Mol Genet.

[B10] Johnston JA, Dalton MJ, Gurney ME, Kopito RR (2000). Formation of high molecular weight complexes of mutant Cu, Zn-superoxide dismutase in a mouse model for familial amyotrophic lateral sclerosis. Proc Natl Acad Sci USA.

[B11] Mishra RS, Bose S, Gu Y, Li R, Singh N (2003). Aggresome formation by mutant prion proteins: the unfolding role of proteasomes in familial prion disorders. J Alzheimers Dis.

[B12] Grenier C, Bissonnette C, Volkov L, Roucou X (2006). Molecular morphology and toxicity of cytoplasmic prion protein aggregates in neuronal and non-neuronal cells. J Neurochem.

[B13] Johnston JA, Ward CL, Kopito R (1998). Aggresomes: a cellular response to misfolded proteins. J Cell Biol.

[B14] Kristiansen M, Messenger MJ, Klohn PC, Brandner S, Wadsworth JD, Collinge J, Tabrizi SJ (2005). Disease-related prion protein forms aggresomes in neuronal cells leading to caspase activation and apoptosis. J Biol Chem.

[B15] McNaught KS, Shashidharan P, Perl DP, Jenner P, Olanow CW (2002). Aggresome-related biogenesis of Lewy bodies. Eur J Neurosci.

[B16] Olanow CW, Perl DP, DeMartino GN, McNaught KS (2004). Lewy-body formation is an aggresome-related process: a hypothesis. Lancet Neurol.

[B17] García-Mata R, Bebök Z, Sorscher EJ, Sztul ES (1999). Characterization and dynamics of aggresome formation by a cytosolic GFP-chimera. J Cell Biol.

[B18] Kawaguchi Y, Kovacs JJ, McLaurin A, Vance JM, Ito A, Yao TP (2003). The deacetylase HDAC6 regulates aggresome formation and cell viability in response to misfolded protein stress. Cell.

[B19] Su GH, Sohn TA, Ryu B, Kern SE (2000). A novel histone deacetylase inhibitor identified by high-throughput transcriptional screening of a compound library. Cancer Res.

[B20] Haggarty SJ, Koeller KM, Wong JC, Grozinger CM, Schreiber SL (2003). Domain-selective small-molecule inhibitor of histone deacetylase 6 (HDAC6)-mediated tubulin deacetylation. Proc Natl Acad Sci USA.

[B21] Baird GS, Zacharias DA, Tsien RY (2000). Biochemistry, mutagenesis, and oligomerization of DsRed, a red fluorescent protein from coral. Proc Natl Acad Sci USA.

[B22] Shaner NC, Campbell RE, Steinbach PA, Giepmans BN, Palmer AE, Tsien RY (2004). Improved monomeric red, orange and yellow fluorescent proteins derived from Discosoma sp. red fluorescent protein. Nat Biotechnol.

[B23] Goggin K, Beaudoin S, Grenier C, Brown AA, Roucou X (2008). Prion protein aggresomes are poly(A)^+ ^ribonucleoprotein complexes that induce a PKR-mediated deficient cell stress response. Biochim Biophys Acta.

[B24] Garcia-Mata R, Bebok Z, Sorscher EJ, Sztul ES (1999). Characterization and dynamics of aggresome formation by a cytosolic GFP-chimera. J Cell Biol.

[B25] Lehotzky A, Tirian L, Tokesi N, Lenart P, Szabo B, Kovacs J, Ovadi J (2004). Dynamic targeting of microtubules by TPPP/p25 affects cell survival. J Cell Sci.

[B26] Araujo FD, Stracker TH, Carson CT, Lee DV, Weitzman MD (2005). Adenovirus type 5 E4orf3 protein targets the Mre11 complex to cytoplasmic aggresomes. J Virol.

[B27] Tomai E, Butz K, Lohrey C, von Weizsacker F, Zentgraf H, Hoppe-Seyler F (2006). Peptide aptamer-mediated inhibition of target proteins by sequestration into aggresomes. J Biol Chem.

[B28] Rajan RS, Illing ME, Bence NF, Kopito RR (2003). Specificity in intracellular protein aggregation and inclusion body formation. Proc Natl Acad Sci USA.

